# Rotational IMRT techniques compared to fixed gantry IMRT and Tomotherapy: multi-institutional planning study for head-and-neck cases

**DOI:** 10.1186/1748-717X-6-20

**Published:** 2011-02-21

**Authors:** Tilo Wiezorek, Tim Brachwitz, Dietmar Georg, Eyck Blank, Irina Fotina, Gregor Habl, Matthias Kretschmer, Gerd Lutters, Henning Salz, Kai Schubert, Daniela Wagner, Thomas G Wendt

**Affiliations:** 1 Department of Radiation Oncology, University of Jena, Jena, Germany; 2 Division of Medical Radiation Physics, Department of Radiotherapy, Medical University Vienna/AKH Wien, Vienna, Austria; 3 Department of Radiation Oncology, Ruppiner Hospitals, Neuruppin, Germany; 4 Department of Radiation Oncology "Praxis Mörkenstrasse", Hamburg, Germany; 5 Department of Radiation Oncology, University of Heidelberg, Germany; 6 Department of Radiation Oncology, Kantonsspital Aarau, Aarau, Switzerland; 7 Department of Radiation Oncology, University of Goettingen, Goettingen, Germany

## Abstract

**Background:**

Recent developments enable to deliver rotational IMRT with standard C-arm gantry based linear accelerators. This upcoming treatment technique was benchmarked in a multi-center treatment planning study against static gantry IMRT and rotational IMRT based on a ring gantry for a complex parotid gland sparing head-and-neck technique.

**Methods:**

Treatment plans were created for 10 patients with head-and-neck tumours (oropharynx, hypopharynx, larynx) using the following treatment planning systems (TPS) for rotational IMRT: Monaco (ELEKTA VMAT solution), Eclipse (Varian RapidArc solution) and HiArt for the helical tomotherapy (Tomotherapy). Planning of static gantry IMRT was performed with KonRad, Pinnacle and Panther DAO based on step&shoot IMRT delivery and Eclipse for sliding window IMRT. The prescribed doses for the high dose PTVs were 65.1Gy or 60.9Gy and for the low dose PTVs 55.8Gy or 52.5Gy dependend on resection status. Plan evaluation was based on target coverage, conformity and homogeneity, DVHs of OARs and the volume of normal tissue receiving more than 5Gy (V_5Gy_). Additionally, the cumulative monitor units (MUs) and treatment times of the different technologies were compared. All evaluation parameters were averaged over all 10 patients for each technique and planning modality.

**Results:**

Depending on IMRT technique and TPS, the mean CI values of all patients ranged from 1.17 to 2.82; and mean HI values varied from 0.05 to 0.10. The mean values of the median doses of the spared parotid were 26.5Gy for RapidArc and 23Gy for VMAT, 14.1Gy for Tomo. For fixed gantry techniques 21Gy was achieved for step&shoot+KonRad, 17.0Gy for step&shoot+Panther DAO, 23.3Gy for step&shoot+Pinnacle and 18.6Gy for sliding window.

V_5Gy _values were lowest for the sliding window IMRT technique (3499 ccm) and largest for RapidArc (5480 ccm). The lowest mean MU value of 408 was achieved by Panther DAO, compared to 1140 for sliding window IMRT.

**Conclusions:**

All IMRT delivery technologies with their associated TPS provide plans with satisfying target coverage while at the same time respecting the defined OAR criteria. Sliding window IMRT, RapidArc and Tomo techniques resulted in better target dose homogeneity compared to VMAT and step&shoot IMRT. Rotational IMRT based on C-arm linacs and Tomotherapy seem to be advantageous with respect to OAR sparing and treatment delivery efficiency, at the cost of higher dose delivered to normal tissues. The overall treatment plan quality using Tomo seems to be better than the other TPS technology combinations.

## Background

Today intensity-modulated radiation therapy (IMRT) is the method of choice for the treatment of patients with complex-shaped planning target volumes (PTV) targets, especially when concave targets are close to a larger number of organs-at-risk (OAR) with different dose constraints and for multiple integrated targets with different dose prescriptions e.g. simultaneous integrated boost (SIB) treatments. The advantage of IMRT for head-and-neck cancer patients is the dose reduction in the parotid glands which implies less xerostomia and therefore has a big impact on the quality of life. Besides all these advantages of IMRT there are some disadvantages too. The delivery of complex plans with traditional IMRT techniques takes extra time and the dose distribution in the PTV is more inhomogeneous compared to conformal techniques. Another important aspect is the higher number of monitor units (MU) in comparison with non-wedged conformal plans. These higher numbers of MUs result in increased peripheral dose, which adds to the generally increased low dose region when applying IMRT [1-3]. Different factors that influence the quality and the complexity of IMRT plans have been investigated by various authors [4-10].

Furthermore, there are some extra requirements for the delivery of IMRT, for instance the high mechanical and dosimetric accuracy of the treatment machine and a TPS with a powerful optimisation and segmentation algorithm.

During the last years new rotational IMRT treatment technologies have become available. These technologies utilize a higher number of degrees of freedom for dose sculpting, i.e. the beam is on during gantry rotation, and at the same time gantry speed, leaf positions, leaf speed and dose rate may be varied. Helical tomotherapy (HT) (Tomotherapy) and rotational IMRT techniques like volumetric-modulated arc therapy (VMAT/Elekta) or RapidArc (Varian) are the most prominent examples. These new technologies enable to achieve treatment plans of similar or better quality compared to static IMRT [11-25]. VMAT and RapidArc can be delivered with standard C-arm gantry linacs. Several authors investigated the plan quality and other parameters in comparisons of these new IMRT modalities with HT or standard IMRT with fixed gantry angles.

Although several papers were published on comparing static with rotational IMRT, they were limited mostly to two treatment planning systems and were usually performed in one institution, i.e. they were limited by planning traditions. To overcome this limitation it was the aim of the present study to benchmark as many upcoming rotational IMRT techniques as possible against a wide range of commonly practised static IMRT and dynamic IMRT techniques using one of the most complex treatment situations in today's clinical practice, a parotid gland sparing head-and-neck technique with simultaneous integrated boost (SIB). The influence of different optimisation algorithms (3 different algorithms for step&shoot) was integral part of this multi-institutional study, but the influence of the dose calculation algorithms was not taken into account for current comparison.

## Methods

### Patients

Ten patients with complex shaped targets in the head-and-neck region (orpharynx, hypopharynx, larynx) suitable for an SIB technique were selected for this retrospective multi-centre treatment planning study. The characteristics of these patients are shown in Table 1.

**Table 1:** Overview of the patients

Patient Nr	Cancer type	TNM stage	Volume PTV1	Volume PTV2	Type
			**in ccm**	**in ccm**	

Patient 1	Oropharynx-Ca	pT4 N1cM0	169	617	Postoperative RT

Patient 2	Hypopharynx-Ca	cT3 cN2a M0	327	989	Primary RT

Patient 3	Larynx-Ca	T4 N2c M0	200	1568	Primary RT

Patient 4	Oropharynx-Ca	pT4 pN2a M0	145	408	Postoperative RT

Patient 5	Oropharynx-Ca	pT4a pN1 cM0	164	709	Postoperative RT

Patient 6	Oropharynx-Ca	T3 N1 M0	279	881	Primary RT

Patient 7	Oropharynx-Ca	pT4 N3 M0	166	768	Primary RT

Patient 8	Larynx-Ca	pT3 N2 M0	151	851	Primary RT

Patient 9	Oropharynx-Ca	cT4cN3 M0	338	850	Primary RT

Patient 10	Oropharynx-Ca	pT4 pN2c cM0	235	1577	Postoperative RT

### Treatment techniques

All PTVs and OARs were contoured in one TPS at the study coordination centre in Jena. CT data including structure sets of all patients were transferred to different centres which provided one of the following treatment technologies: Tomotherapy, VMAT, RapidArc, sliding window and step&shoot IMRT. More specifically, the following TPS were used: the TPS HiArt (Tomotherapy) was used for the helical tomotherapy (Tomo); rotational IMRT (VMAT) for an ELEKTA linac was planned with the TPS Monaco while rotational IMRT performed with a Varian linac (RadpidArc) was planned with Eclipse. For the static gantry IMRT four TPS were used: for step&shoot IMRT the KonRad (Siemens) system, the TPS Pinacle (ADAC) and the Panther DAO (Prowess), and finally for sliding window IMRT the Eclipse (Varian) system. All treatment plans were calculated with a nominal energy of 6 MV. The detailed overview about the used technologies, the TPS, linac e.t.c. is shown in table 2.

**Table 2:** Overview of used technologies, TPS and versions, linacs, number of beams or arcs and energy

technology	TPS	version	linear accelerator	number of arcs/beams	energy	algorithm
S&S	Konrad	2.2.23	Siemens Oncor	11 beams	6 MV	Pencil Beam

S&S	Panther DAO	4.71	Siemens Artiste	11 beams	6 MV	Pencil Beam

S&S	Pinnacle	8.0 m	Siemens Oncor	11 beams	6 MV	Pencil Beam

SW	Eclipse	8.1	Varian Clinac 1600	7 beams	6 MV	Pencil Beam

VMAT	Monaco	2.0.1	Elekta Synergy MLCi	2 arcs	6 MV	Monte Carlo XVMC

Tomotherapy	Hi-Art	3.1.4.7	Tomotherapy Hi-Art	-------------	6 MV	collapsed cone

Rapid Arc	Eclipse	8.9	Varian Clinac 2300	2 arcs	6 MV	AAA

The aim of the planning study was to achieve similar median doses in the PTVs for all ten patients. Dependent on the therapy concept which is based on the status of resection, the prescribed median PTV dose was defined as 52.2Gy or 55.8Gy to the lymph node region (PTV2) and as 60.9Gy or 65.1Gy to the integrated boost volume (PTV1). The minimal criterium (93% of the prescribed dose to minimal 99% of the PTV) was deduced from the RTOG H0022 protocol. The maximum dose criterion was defined as maximal 1% of the PTV receives maximal 110%. Additionally, the OAR objective for the parotid glands (D_median _< 26Gy), for the mandibular (D_median _< 45Gy) and the spinal cord plus a 7 mm margin (D_max _< 43Gy) should be satisfied. Fulfilling of the dose criteria for the PTV is given highest priority for treatment planning, except the criteria for the spinal cord could not be met.

### Treatment plan evaluation

All doses in the evaluation are relative doses, normalised to the prescribed doses of PTV1 and PTV2. The evaluation was based on several criteria. The first criterium was the PTV coverage with 93% of the prescribed dose. The conformation of the PTVs (with respect to 93% of the prescribed dose) was described by the conformity index (CI = Volume_93%_/PTV). This specific formula was selected based on the assumption that no more than 1% of any PTV should receive <93% of its prescribed dose as minimum criteria, i.e. almost 100% of the PTV should received at least 93% of the dose. Target dose heterogeneity was described by the homogeneity index (HI=[D_5%_-D_95%_]/D_mean_), i.e. a small HI indicates a better plan in the comparison. Another main focus of the comparison was put on the DVHs of the OARs and the volume of healthy tissue receiving more than 5Gy (V_5Gy_). Finally, the cumulative monitor units (MUs) and treatment times of the different technologies were compared. For that purpose the different linac calibrations conditions were normalised except the Tomotherapy machine.

All evaluation parameters were averaged over the 10 patients for each technique and planning modality. The standard deviations for all evaluation values were calculated over the ten patients.

### Results

**Figure 1:**
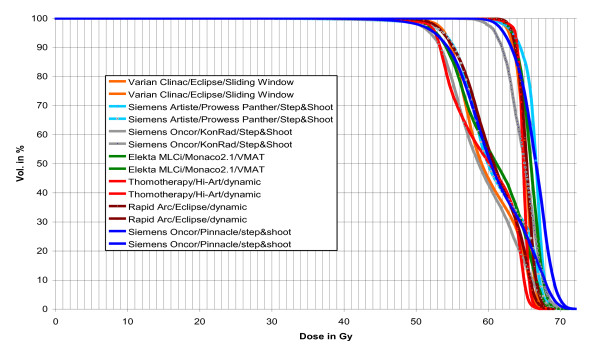
**The prescribed doses are 55.8 Gy to the low dose region and 65.1Gy to the high dose region**. The PTV2 is a subset of PTV1.

**Figure 2:**
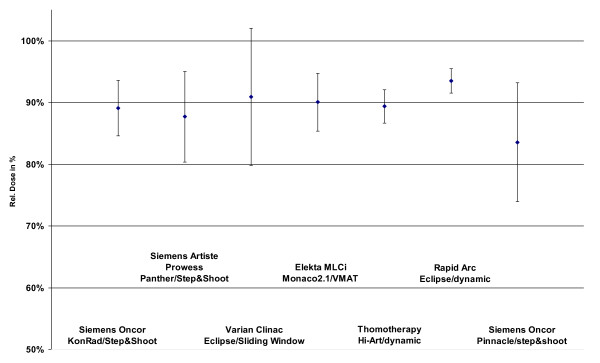
**Dose at 99% of the PTV2 dependend on technology and TPS**.

**Figure 3:**
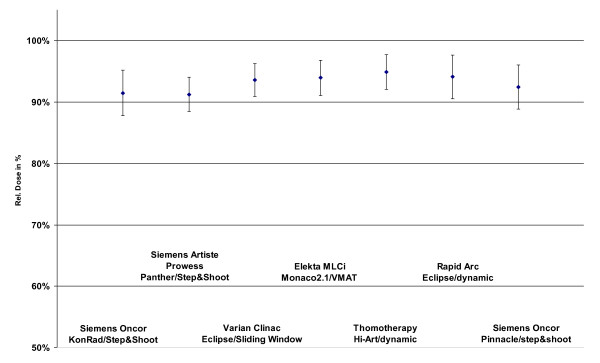
**Dose at 99% of the PTV1 dependend on technology and TPS**.

All IMRT technologies with their respective TPSs were able to provide treatment plans which fulfilled the planning goals. Figure 1 shows as an example DVHs for one patient for both PTVs and all IMRT techniques. The coverage of the PTVs is seen in figure 2 and 3. In that figures the doses which is given to 99% of the PTVs is used as criterium. These doses are in a range of 91% till 95% of the prescribed dose for PTV1 and between 84% and 93% for the PTV2.

**Figure 4:**
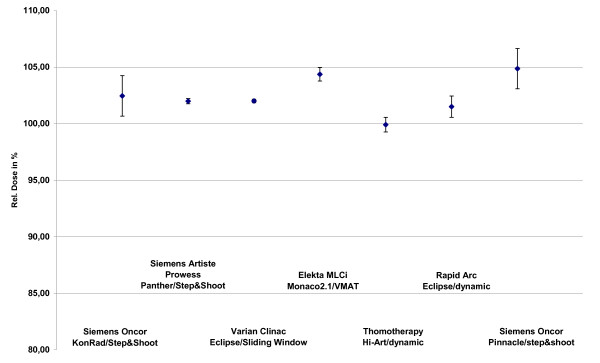
**Median doses of the PTV2 dependend on technologie and TPS**.

**Figure 5:**
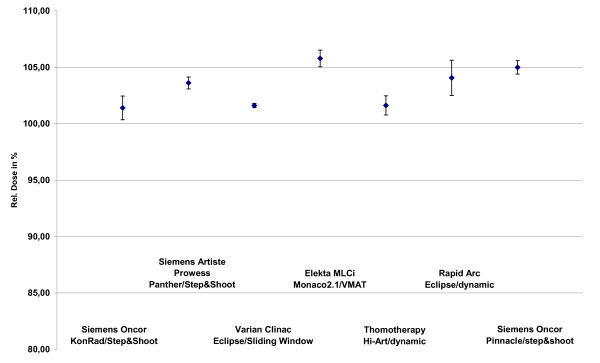
**Median doses of the PTV1 dependend on technologie and TPS**.

The median doses of the low and high dose PTVs are in a range of 99.9% (Tomo) and 104.9% (VMAT) for PTV2 and between 101.4% (Konrad) and 105.8% (VMAT) for PTV1 as seen in figure 4 and figure 5.

### Conformation evaluation

**Figure 6:**
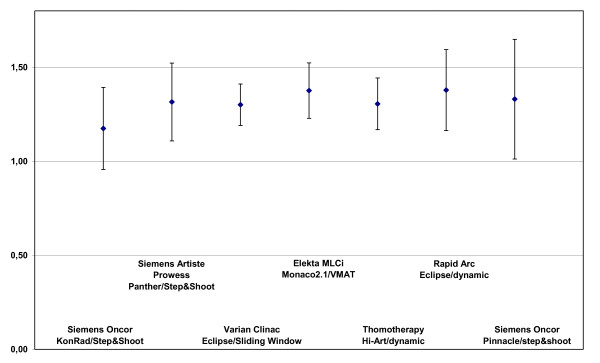
**Conformity index of the PTV2 dependend on technologie and TPS**.

**Figure 7:**
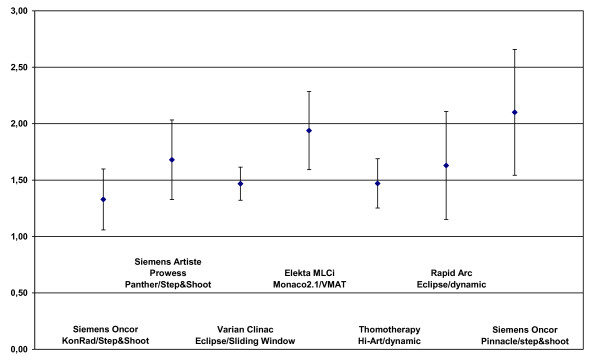
**Conformity index of the PTV1 dependend on technologie and TPS**.

Figure 6 and figure 7 show the CI values. The best conformation was achieved with the KonRad+step&shoot with a mean CI of 1.17 for the PTV2. The CI values of the PTV2 were rather similar with 1.30 for sliding window, 1.31. for Tomo, 1.32 for DAO+step&shoot and 1.33 for Pinacle+step&shoot, while it was 1.38 for both VMAT and RapidArc.

The conformation of the PTV1 was again best for KonRad+Step&shoot (1.33). The second best result was achieved by the sliding window technique and Tomo (both 1.47), followed by RapiArc (1.63), DAO+step&shoot (1.68), VMAT (1.94) and Pinacle+step&shoot only with 2.82.

### Homogeneity evaluation

**Figure 8:**
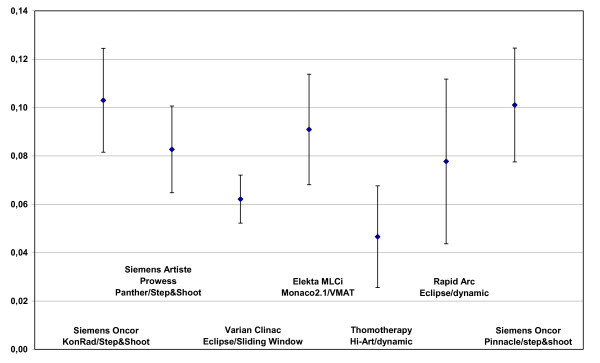
**Homogeneity index of the PTV1 dependend on technologie and TPS**.

The HI values for PTV2 were not evaluated because not all TPS were able to provide PTV2 excluded the Boost PTV. HI values for PTV1 are shown in figure 8. The best HI for the PTV1 was found with Tomo (0.047), followed by sliding window (0.062). Higher HI values were found for RapidArc (0.078) and DAO+step&shoot (0.083), VMAT (0.091) treatment plans, as well as for KonRad+step&shoot and Pinacle+step&shoot plans (both 0.100).

### Evaluation of OAR sparing

A summary of the results concerning OAR sparing is shown in table 3. Not all TPS could reach the OAR objectives. The median doses of the parotids were 14.1Gy for Tomo, 17.0 Gy for step&shoot+DAO, 18.6Gy for sliding window, 21Gy for step&shoot+KonRad, 23Gy for VMAT, 23.3 Gy for step&shoot+Pinnacle and 26.5.Gy for RapidArc.

**Table 3:** OAR doses dependend on IMRT technology

	KonRad/S&S	Panther DAO/S&S	Eclipse/SW	VMAT	Tomotherapy	Rapid Arc	Pinnacle/S&S
**myelon max.dose/Gy**	42.34 ± 0.59	42.43 ± 0.50	44.89 ± 3.59	40.64 ± 1.58	34.25 ± 2.69	41.98 ± 0.26	43.17 ± 0.52

**parotides median dose/Gy**	21.01 ± 4.59	17.24 ± 2.97	18.68 ± 4.29	22.98 ± 4.41	14.11 ± 2.37	26.47 ± 5.31	22.46 ± 3.62

**mandible median dose/Gy**	39,99 ± 8,65	42,90 ± 7,19	43,70 ± 8,48	43,12 ± 9,51	36,14 ± 9,77	41,21 ± 8,98	39,50 ± 5,71

The maximal doses to the myelon plus 7 mm margin varied between 34.2Gy (Tomo), 40.6Gy (VMAT), 42 Gy (RapidArc), 42.4 Gy (step&shoot+DAO), 42.9Gy (KonRad+step&shoot), 43.2 Gy (Pinnacle+step&shoot), to 44.9 Gy (sliding window).

The median doses to the mandible were 36.1Gy (Tomo), 39.5 (Pinnacle+step&shoot), 40Gy (KonRad+step&shoot), 41.2Gy (RapidArc), 42.9Gy (step&shoot+DAO), 43.1Gy (VMAT), 43.7Gy (sliding window).

### Evaluation of low dose burden, MUs and treatment time

Table 4 summarized the results of the volume receiving more than 5Gy (V_5Gy_), the MU and treatment time, respectively. The lowest V_5Gy _values were achieved with the sliding window technique with fixed gantry angles (3499 ccm). The other technologies present the following values in increasing order: VMAT (4498 ccm), KonRad+step&shoot (4525 ccm), Pinacle+step&shoot (5010 ccm), Tomo (5122 ccm), DAO+step&shoot (5332 ccm) and RapidArc (5480 ccm).

**Table 4:** MUs, treatment time, V_5Gy _dependend on IMRT technology

	KonRad/S&S	Panther DAO/S&S	Eclipse/SW	VMAT	Tomotherapy	Rapid Arc	Pinnacle/S&S
MU normalised	800.44 ± 100.90	408.27 ± 17.97	1139.86 ± 239.45	500.82 ± 71.59	×	436.92 ± 36.53	1059.63 ± 134.85

treatment time/min	11.18 ± 2.64	7.07 ± 0.72	10.5 ± 1.00	11.8 ± 1.44	7.74 ± 0.80	2.48 ± 0.01	11 ± 0.45

Volume/ccm	4524.94 ± 1969.67	5331.76 ± 1437.55	3802.11 ± 899.31	4497.85 ± 1196.30	5122.01 ± 1647.57	5479.37 ± 1524.97	5010.46 ± 1149.93
							
receiving >5 Gy							

The comparison of the MUs for the different technologies showed a wide range. The normalised MUs were lowest for DAO+step&shoot (408), followed by RapidArc (437) and VMAT (501). The step&shoot technique planned with KonRad required on average 800 MU, but when planned with Pinnacle it increased up to 1059 MU on average. The sliding window technique needs on average 1140 MU for IMRT delivery.

The shortest mean treatment times were associated with RapidArc (2.5 min with 2 arcs), followed by DAO+step&shoot (7 min), Tomo (8 min), VMAT (9 min with 2 arcs), sliding window (10.5 min) and step&shoot with KonRad and Pinnacle (11 min).

## Discussion

The present study is a multi-institutional study; this implies that there are some "subjective" factors depending on planning philosophy of the respective hospital e.g. number of beam directions, number of segments and arcs, limitations of the MLCs, weighting of the importance of PTV and OAR. Another role plays the level of experience of the planners in the different centres that's why we selected for every technology and TPS combination experienced users. But in the last consequence the results of this multi-institutional study show that all used IMRT technologies together with their TPSs have the power to provide treatment plans with a satisfying target coverage while at the same time respecting the defined OAR criteria. At least there is no best technology with respect to all evaluation parameters, i.e. all techniques are connected with some advantages and with some disadvantages. As far as treatment planning is concerned, there were substantial differences in terms of usability to specify the planning goals for the different volumes. It would be of great help for treatment planning if functions where available in TPS that excluded intersections automatically or where priorities to different PTVs with intersections could be assigned.

The results are in good agreement with published data [26-29] regarding the volumatric arc therapy. Only the results of our study getting with sliding window are much better than in [17]. A differentiation of the patients in the two groups (post-operative patients and primary RT) did not show significant differences in the results.

All treatment plans offer a very good coverage of the PTV1 and a good coverage of the PTV2. The lowest dose to the PTV2 with clearly inferior results compared to the other techniques was achieved with the Pinnacle step&shoot combination. The median doses for the PTV2 and the PTV1 were in a range between 100% and 106%. This implies that the planners of the participating institutes improved the coverage of the PTVs with the help of an increase of the median dose. The requirements demanded by the HR0022 protocol are more or less fulfilled. ICRU recommendations for prescribing, reporting and recording IMRT have just been which will be helpful in the future to harmonize IMRT practice [30].

Sliding window, RapidArc and Tomo techniques resulted in better target dose homogeneity for the PTV1 compared to VMAT and step&shoot with Panther DAO, Pinnacle and KonRad.

All technologies TPS combinations fulfill the OAR constrains. Only the high myelon maximal dose receiving with sliding window is demonstrative (but with a margin of 7 mm clinically acceptable). The highest median dose to the spared parotid while using the RapiArc is peculiar too.

The volume which receives equal or more than 5Gy is lowest with the sliding window technique (3800 ccm), followed by the VMAT and KonRad step&shoot (about 4500 ccm). Pinnacle step&shoot, Tomo, Panther DAO and RapidArc deliver doses of equal or more than 5Gy to volumes of 5000 ccm or bigger. It is of interest that neither the "classic IMRT" with fixed gantry angles nor the rotation based IMRT is clearly the superior solution. It seems that rotational IMRT techniques do not automatically generate more volume that receives dose of equal or more than 5Gy. The volume could probably be even further reduced using higher photon beam energies.

The treatment delivery times obtained in the present study were shortest for the RapidArc solution. The delivery times for Tomo and Panther DAO were in the medium range while VMAT, step&shoot with Konrad or Pinnacle and with sliding window were characterised by the longest ones. As far as the VMAT results on delivery efficiency are concerned, it needs to be emphasized that Monaco Version 2.01 was used in the present study, which was improved recently with a new sequencer available in successive versions of this TPS.

The MUs are significantly reduced for the DAO step&shoot (408MU), RapidArc (437MU) and VMAT (501MU). The MUs needed for a step&shoot KonRad plan is situated in the centre (about 800MU). Pinnacle step&shoot needs 1060MU and sliding window takes the highest number of 1140MU. It is known that the number of MU is one factor which influences the peripheral dose, but there are some other factors like the linac head shielding and collimation system (shape, thickness, material), the focus body distance and the spectrum of the beam. The peripheral dose is of importance without any doubt but in the particular case subordinated relativ to the treatment plan quality.

## Conclusions

This is the first multi-institutional study that determined the influence of seven different combinations of treatment technologies and TPS combinations for the planning of head and neck cancer treatments for a simultaneous integrated boost technique. The results presented above indicate that all IMRT delivery technologies with their associated TPS provide IMRT plans with satisfying target coverage while at the same time mostly respecting the defined OAR criteria.

Sliding window, RapidArc and Tomo techniques provide better target dose homogeneity compared to VMAT and step&shoot with Panther DAO, Pinacle and KonRad. The conformity reached was best for KonRad for high and low dose PTV with a remarkable distance to the all other IMRT techniques. The overall treatment plan quality using Tomo regarding target coverage, HI, CI and OAR sparing seems to be better than the other TPS technology combinations. For the parotid gland clear median dose differences were observed for the different IMRT techniques. Rotational IMRT and Tomo seem to be advantageous with respect to OAR sparing sometimes and treatment delivery efficiency, at the cost of higher dose burden (>5Gy) to normal tissues. The application times are shortest for RapidArc with some concessives e.g. parotid sparing. The combination of Panther DAO and step&shoot shows that a segmentation algorithm which is optimised for time saving applications reduces the treatment time with plan quality concessions too. The applications need the most time with VMAT, with step&shoot with Konrad or Pinacle and with sliding window.

We expect a medical relevance of the results of our study e.g. partial underdosage, different OAR sparing, dose burden with 5Gy or more; but this should be investigated in prospective studies.

## Competing interests

The authors declare that they have no competing interests.

## Authors' contributions

TW coordinated the entire study. Patient accrual and clinical data collection was done by TGW. Treatment planning was conducted by TW, EB, IF, GH, MK, GL, KS, HS, DW.

Data collection was worked out by TB. Data analysis was done by TW and TB.

The manuscript was prepared by TW. Corrections and/or improvements were suggested by DG, IF, HS, KS and TGW. Major revisions were done by TW. All authors read and approved the final manuscript.

## References

[B1] Bennett BR Lamba MA Elson HR Analysis of peripheral doses for base of tongue treatment by linear accelerator and helical TomoTherapy IMRT J Appl Clin Med Phys 2010 11 3 31 36 10.1120/jacmp.v11i3.3136 PMC5720425 20717081

[B2] Wiezorek T Schwahofer A Schubert K The influence of different IMRT techniques on the peripheral dose: a comparison between sMLM-IMRT and helical tomotherapy Strahlenther Onkol 2009 185 10 696 702 10.1007/s00066-009-2005-9 19806336

[B3] Wiezorek T Georg D Schwedas M Salz H Wendt TG Experimental determination of peripheral photon dose components for different IMRT techniques and linear accelerators Z Med Phys 2009 19 2 120 8 19678527 10.1016/j.zemedi.2009.01.008

[B4] Georg D Kroupa B Georg P Winkler P Bogner J Dieckmann K Pötter R Inverse planning - a comparative intersystem and interpatient constraint study Strahlenther Onkol 2006 182 8 473 80 10.1007/s00066-006-1531-y 16896594

[B5] Georg P Georg D Hillbrand M Kirisits C Pötter R Factors influencing bowel sparing in intensity modulated whole pelvic radiotherapy for gynaecological malignancies Radiother Oncol 2006 80 1 19 26 Epub 2006 Jun 12 10.1016/j.radonc.2006.04.014 16766068

[B6] Hunt MA Jackson A Narayana A Lee N Geometric factors influencing dosimetric sparing of the parotid glands using IMRT Int J Radiat Oncol Biol Phys 2006 66 1 296 304 10.1016/j.ijrobp.2006.05.028 16904529

[B7] Luan S Wang C Cao D Leaf-sequencing for intensity-modulated arc therapy using graph algorithms Med Phys 2008 35 61 69 10.1118/1.2818731 18293562

[B8] Shepard DM Earl MA Li XA Direct aperture optimization: A turnkey solution for step-and-shoot IMRT Med Phys 2002 29 1007 1018 10.1118/1.1477415 12094970

[B9] Søndergaard J Høyer M Petersen JB Wright P Grau C Muren LP The normal tissue sparing obtained with simultaneous treatment of pelvic lymph nodes and bladder using intensity-modulated radiotherapy Acta Oncol 2009 48 2 238 44 18759144 10.1080/02841860802251575

[B10] Ulrich S Nill S Oelfke U Development of an optimization concept for arc-modulated cone beam therapy Phys Med Biol 2007 52 4099 4119 10.1088/0031-9155/52/14/006 17664597

[B11] Cameron C Sweeping-window arc therapy: An implementation of rotational IMRT with automatic beam-weight calculation Phys Med Biol 2005 50 4317 4336 10.1088/0031-9155/50/18/006 16148396

[B12] Ansari DO Esiashvili N Dhabaan AH Jarrio CS Elder ES Crowder M Koontz-Raisig W Shu HG Is Intensity Modulated Arc Therapy (IMAT) Better Than Non-rotational Intensity Modulated Radiation Therapy (IMRT) for Pediatric Brain Tumors? Int J Radiat Oncol Biol Phys 2009 75 3 510

[B13] Cao D Holmes TW Afghan MKN Comparison of plan quality provided by intensity-modulated arc therapy and helical tomotherapy Int J Rad Oncol Biol Phys 2007 69 240 250 10.1016/j.ijrobp.2007.04.073 17707278

[B14] Clivio A Fogliata A Franzetti-Pellanda A Nicolini G Vanetti E Wyttenbach R Cozzi L Volumetric-modulated arc radiotherapy for carcinomas of the anal canal: A treatment planning comparison with fixed field IMRT Radiother Oncol 2009 92 1 118 124 10.1016/j.radonc.2008.12.020 19181409

[B15] Cozzi L Dinshaw KA Shrivastava SK Mahantshetty U Engineer R Deshpande DD Jamema SV Vanetti E Clivio A Nicolini G Fogliata A A treatment planning study comparing volumetric arc modulation with RapidArc and fixed field IMRT for cervix uteri radiotherapy Radiother Oncol 2008 89 2 180 191 10.1016/j.radonc.2008.06.013 18692929

[B16] Doornaert P Verbakel WF Bieker M Slotman BJ Senan S RapidArc Planning and Delivery in Patients with Locally Advanced head-and-neck Cancer Undergoing Chemoradiotherapy Int J Radiat Oncol Biol Phys 2011 79 2 429 435 10.1016/j.ijrobp.2009.11.014 20421159

[B17] Jacob V Bayer W Astner ST Busch R Kneschaurek P A Planning Comparison of Dynamic IMRT for Different Collimator Leaf Thicknesses with Helical Tomotherapy and RapidArc for Prostate and Head and Neck Tumors Strahlenther Onkol 2010 186 9 502 510 Epub 2010 Aug 30 10.1007/s00066-010-2124-3 20803184

[B18] Otto K Volumetric modulated arc therapy: IMRT in a single gantry arc Med Phys 2007 35 310 317 10.1118/1.2818738 18293586

[B19] Sheng K Molloy J Read PW Intensity-modulated radiation therapy (IMRT) dosimetry of the head and neck: a comparison of treatment plans using linear accelerator-based IMRT and helical tomotherapy Int J Radiat Oncol Biol Phys 2006 65 917 23 10.1016/j.ijrobp.2006.02.038 16751074

[B20] Wang C Luan S Tang G Arc-modulated radiation therapy (AMRT): A single-arc form of intensity-modulated arc therapy Phys Med Biol 53 2008 6291 6303 18936519 10.1088/0031-9155/53/22/002

[B21] Wieland P Dobler B Mai S Hermann B Tiefenbacher U Steil V Wenz F Lohr F IMRT for postoperative treatment of gastric cancer: covering large target volumes in the upper abdomen: a comparison of a step-and-shoot and an arc therapy approach Int J Radiat Oncol Biol Phys 2004 59 4 1236 1244 10.1016/j.ijrobp.2004.02.051 15234061

[B22] Wolff D Stieler F Welzel G Lorenz F Abo-Madyan Y Mai S Herskind C Polednik M Steil V Wenz F Lohr F Volumetric modulated arc therapy (VMAT) vs. serial tomotherapy, step-and-shoot IMRT and 3D-conformal RT for treatment of prostate cancer Radiother Oncol 2009 93 2 226 233 10.1016/j.radonc.2009.08.011 19765846

[B23] Wolff U Stieler F Abo-Madyan Y Polednik M Steil V Mai S Wenz F Lohr F Volumetric Intensity Modulated Arc Therapy (VMAT) vs. Serial Tomotherapy and Segmental (Step and Shoot) IMRT for Treatment of Prostate Cancer Int J Radiat Oncol Biol Phys 2008 72 1 56218793958

[B24] Wu Q Kirkpatrick J Yoo S McMahon R Thongphiew D Yin F Comparing Static vs. Rotational IMRT for Spine Body Radiotherapy Int J Radiat Oncol Biol Phys 2009 75 3 672 10.1016/j.ijrobp.2008.11.057 19733447

[B25] Yu CX Intensity-modulated arc therapy with dynamic multileaf collimation: an alternative to tomotherapy Phys Med Biol 1995 40 1435 1449 10.1088/0031-9155/40/9/004 8532757

[B26] Sterzing F Sroka-Perez G Schubert K Münter MW Thieke C Huber P Debus J Herfarth KK Evaluating target coverage and normal tissue sparing in the adjuvant radiotherapy of malignant pleural mesothelioma: Helical tomotherapy compared with step-and-shoot IMRT Radiother Oncol 2008 86 2 251 257 10.1016/j.radonc.2007.12.010 18207597

[B27] Tang G Earl MA Luan S Converting multiple-arc intensity-modulated arc therapy into a single arc for efficient delivery Int J Radiat Oncol Biol Phys 2007 69 S673

[B28] Vanetti E Clivio A Nicolini G Fogliata A Ghosh-Laskar S Agarwal JP Upreti RR Budrukkar A Murthy V Deshpande DD Shrivastava SK Dinshaw KA Cozzi L Volumetric modulated arc radiotherapy for carcinomas of the oro-pharynx, hypo-pharynx and larynx: a treatment planning comparison with fixed field IMRT Radiother Oncol 2009 92 1 111 7 10.1016/j.radonc.2008.12.008 19157609

[B29] Verbakel WF Cuijpers JP Hoffmans D Bieker M Slotman BJ Senan S Volumetric intensity-modulated arc therapy vs. conventional IMRT in head-and-neck cancer: a comparative planning and dosimetric study Int J Radiat Oncol Biol Phys 2009 74 1 252 9 10.1016/j.ijrobp.2008.12.033 19362244

[B30] ICRU-Report 83: Prescribing, Recording and Reporting Intensity-Modulated Photon-Beam Therapy (IMRT)

